# P02-025 - Homozygous Q705K sequence variant in NLRP3

**DOI:** 10.1186/1546-0096-11-S1-A132

**Published:** 2013-11-08

**Authors:** S Berg, M Sundqvist, K Christenson, A Welin, H Björnsdottir, J Bylund, P Wekell, P Söderkvist, A Karlsson

**Affiliations:** 1 The Queen Silvia Childrens Hospital, Sahlgrenska University Hospital, Göteborg, Sweden; 2 Pediatrics, Gothenburg University, Gothenburg, Sweden; 3 Department of Rheumatology and Inflammation Research, Gothenburg University, Gothenburg, Sweden; 4 The Queen Silvia Childrens Hospital, Sahlgrenska University Hospital, Gothenburg, Sweden; 5 Pediatrics, NU-Hospital Organization, Uddevalla, Sweden; 6 Division of Cellbiology, Linköping University, Linköping, Sweden

## Introduction

The *NLRP3* sequence variant Q705K (Q703K) might have an increased frequency in autoinflammatory conditions including PFAPA and atypical cases of CAPS. It has also been discussed as a modifier of inflammation in other inflammatory conditions. We report the clinical picture and laboratory findings in a patient with the homozygous sequence variant Q705K in *NLRP3.*

## Case report

The patient, a 12-year-old boy with healthy parents, experienced his first long febrile episode, associated with abdominal pain, aseptic meningitis, spleenitis and increased inflammatory markers, at the age of 2.5 years. The patient was given corticosteroids, and responded well. After the age of three years, the patient developed recurrent febrile episodes (approximately twice a year) with abdominal pain of a duration exceeding 2 weeks, without, skin rash and conjunctivitis. Corticosteroid treatment was necessary to terminate the episodes. As steroids were given early during the episodes, the full clinical picture is not known, except for that the patient developed another aseptic meningitis. The severity of the episodes increased and today the patient depends on continuous steroid treatment. Anakinra and colchicine has been used without satisfactory response.

During febrile episodes, inflammatory markers including SAA were elevated. Also, IL18 was increased while IgD and IgA were normal.

The genes *TNFSR1A* and *NLRP3* were screened and a homozygous sequence variant, Q705K, was found in *NLRP3*.

We have investigated cellular and molecular features after the patient had developed fever, abdominal pain and increased inflammatory markers, due to partial withdrawal of corticosteroids. Analysing secretion of cytokines by isolated monocytes incubated for 20 h, in the absence and presence of LPS, we found that the patient produced less IL10 as compared to parents/controls and that the production of IP10 was decreased in both the patient and the parents as compared to controls. The production of IL1β and the monocyte caspase-1 activity were similar between the patient and parents/controls. Also, the production of IL6, IL8, IL12, IL17, GMCSF, INFγ and TNFα were similar. Neutrophil functions, including apoptosis, degranulation and ROS production, were normal.

## Discussion

The sequence variant Q705K in *NLRP3* has been considered a polymorphism in its heterozygous form but might also be a low-penetrance mutation. To our knowledge this is the first description of a patient with a severe inflammatory condition and a homozygote sequence variant Q705K. The patient did not respond to IL1β blockade in a moderate dose and IL1β mediation could not be confirmed in our laboratory investigation. However, the clinical unresponsiveness to IL1β blockade could be related to the dose administered and the corticosteroids may have influenced the laboratory results. It is probable that the homozygous Q705K variant contributes to this severe inflammatory condition in a multifactorial fashion, as the phenotype otherwise would be much more common than clinically found in Sweden.

## Disclosure of interest

None declared.

